# Changes in salivary microbiota due to gastric cancer resection and its relation to gastric fluid microbiota

**DOI:** 10.1038/s41598-023-43108-8

**Published:** 2023-09-22

**Authors:** Eri Komori, Nahoko Kato-Kogoe, Yoshiro Imai, Shoichi Sakaguchi, Kohei Taniguchi, Michi Omori, Mayu Ohmichi, Shota Nakamura, Takashi Nakano, Sang-Woong Lee, Takaaki Ueno

**Affiliations:** 1 Department of Dentistry and Oral Surgery, Faculty of Medicine, Osaka Medical and Pharmaceutical University, 2-7 Daigaku-machi, Takatsuki City, Osaka 569-8686 Japan; 2 Department of General and Gastroenterological Surgery, Faculty of Medicine, Osaka Medical and Pharmaceutical University, 2-7 Daigaku-machi, Takatsuki City, Osaka 569-8686 Japan; 3 Department of Microbiology and Infection Control, Faculty of Medicine, Osaka Medical and Pharmaceutical University, 2-7 Daigaku-machi, Takatsuki City, Osaka 569-8686 Japan; 4 Translational Research Program, Osaka Medical and Pharmaceutical University, 2-7 Daigaku-machi, Takatsuki City, Osaka 569-8686 Japan; 5 Department of Infection Metagenomics, Genome Information Research Center, Research Institute for Microbial Diseases, Osaka University, Suita, Japan

**Keywords:** Cancer, Microbiology, Gastroenterology, Health care, Medical research

## Abstract

Gastric cancer is one of the leading causes of death worldwide, and resections are performed to cure the disease. We have previously reported the changes in the gastric microbiota after gastric cancer resection, which may be associated with the oral microbiota; however, the changes in the oral microbiota remain uncharacterized. This study aimed to characterize the changes in the salivary microbiota caused by gastric cancer resection and to evaluate their association with the gastric fluid microbiota. Saliva and gastric fluid samples were collected from 63 patients who underwent gastrectomy before and after surgery, and a 16S rRNA metagenomic analysis was performed to compare the microbiota composition. The number of bacterial species in the salivary microbiota decreased, and the bacterial composition changed after the resection of gastric cancer. In addition, we identified several bacterial genera that varied significantly in the salivary microbiota, some of which also showed similar changes in the gastric fluid microbiota. These findings indicate that changes in the gastric environment affect the oral microbiota, emphasizing the close association between the oral and gastric fluid microbiota. Our study signifies the importance of focusing on the oral microbiota in the perioperative period of gastrectomy in patients with gastric cancer.

## Introduction

Cancer patients are expected to be 28.4 million worldwide in 2040, a 47% rise from 2020. Gastric cancer is the fifth most common cancer and the fourth leading cause of cancer deaths worldwide^1^. It is a major public health problem, especially in East Asian countries, such as Japan, Korea, and China, where the incidence of gastric cancer is higher than in other countries^2^. The carcinogenesis and progression of gastric cancer are associated with environmental and microbial factors, such as *Helicobacter pylori* (*H. pylori*) infection^3^. *H*. *pylori* generates ammonia, neutralizes gastric acidity, and promotes inflammation, contributing to colonization by other microbes and changing the gastric microenvironment. Therefore, the importance of the gastric microbial environment in gastric cancer has become apparent^4,5^.

In recent years, microbiota, a complex microbial community consisting of an enormous number of bacteria in distinct habitats in the human body, has been found to be intricately involved in health and disease. Next-generation sequencing (NGS) technology and associated bioinformatics tools have facilitated the analysis of the whole microbiome, including microbial species of very low abundance and those that cannot be cultured^6–8^. Studies have revealed that once considered sterile, the stomach is rich in bacteria (the gastric microbiota), and the oral cavity harbors more than 700 species of bacteria. The role of this microbiota in cancer is attracting attention^9^. The microbiota in the gastric mucosa and gastric fluid is vital for gastric homeostasis and is associated with gastric cancer^10–13^. The microbiota in the oral cavity has recently been associated with local oral diseases and systemic diseases^14–16^, including gastric cancer. The bacterial composition in the oral cavity of patients with gastric cancer is significantly different from that of the control group^17^, suggesting that oral microbiota may contribute to gastric cancer and function as diagnostic biomarkers for gastric cancer^18^.

Oral bacteria may influence the gastrointestinal microbiota by translocating into the digestive tube with saliva. For example, certain resident oral bacteria, such as *Fusobacterium nucleatum* and *Porphyromonas gingivalis,* translocate to the gastrointestinal tract and cause various gastrointestinal diseases, such as inflammatory bowel disease and colorectal cancer^19,20^. In the stomach, like in the colon, oral bacteria may affect homeostasis and be associated with inflammation and carcinogenesis^17^. However, how oral bacteria swallowed with saliva pass through the stomach and colonize the gastric mucosa and their role in health and disease are unclear.

Epidemiologically, poor oral health and tooth loss have been demonstrated as possible risk factors for gastric cancer^21,22^. Furthermore, periodontitis, one of the most common oral bacterial infections, is a major risk factor for infectious complications following gastrointestinal surgery, including gastric cancer gastrectomy^23^. These findings suggest that oral bacteria and the resulting inflammation may be associated with both gastric cancer and postoperative complications by affecting the gastric microbiota. Oral interventions may change the gastrointestinal tract's bacterial environment, including that of the stomach^24^; therefore, the importance of oral hygiene in gastric cancer patients is becoming widely recognized. However, there is limited information about the changes in the perioperative oral microbiota, and it needs to be investigated.

We have previously reported changes in the gastric fluid microbiota following gastric cancer resection^25^. Since gastric bacteria are thought to originate from the oral cavity, the oral microbiota may affect the gastric microbiota after gastrectomy. Furthermore, gastrectomy for gastric cancer affects the intestinal microbiota, increasing the number of orally derived bacteria in the gut, and is associated with postoperative complications^26^. Since the oral cavity is connected to the stomach and intestines through the digestive tract, we hypothesized that changes in the gastric environment due to gastric cancer resection might affect not only the gastric and gut microbiota but also the oral microbiota. In this study, we aimed to characterize the changes in the salivary microbiota caused by the resection of gastric cancer and to evaluate their association with the gastric fluid microbiota.

## Results

### Salivary microbiota composition

**Figure 1:**
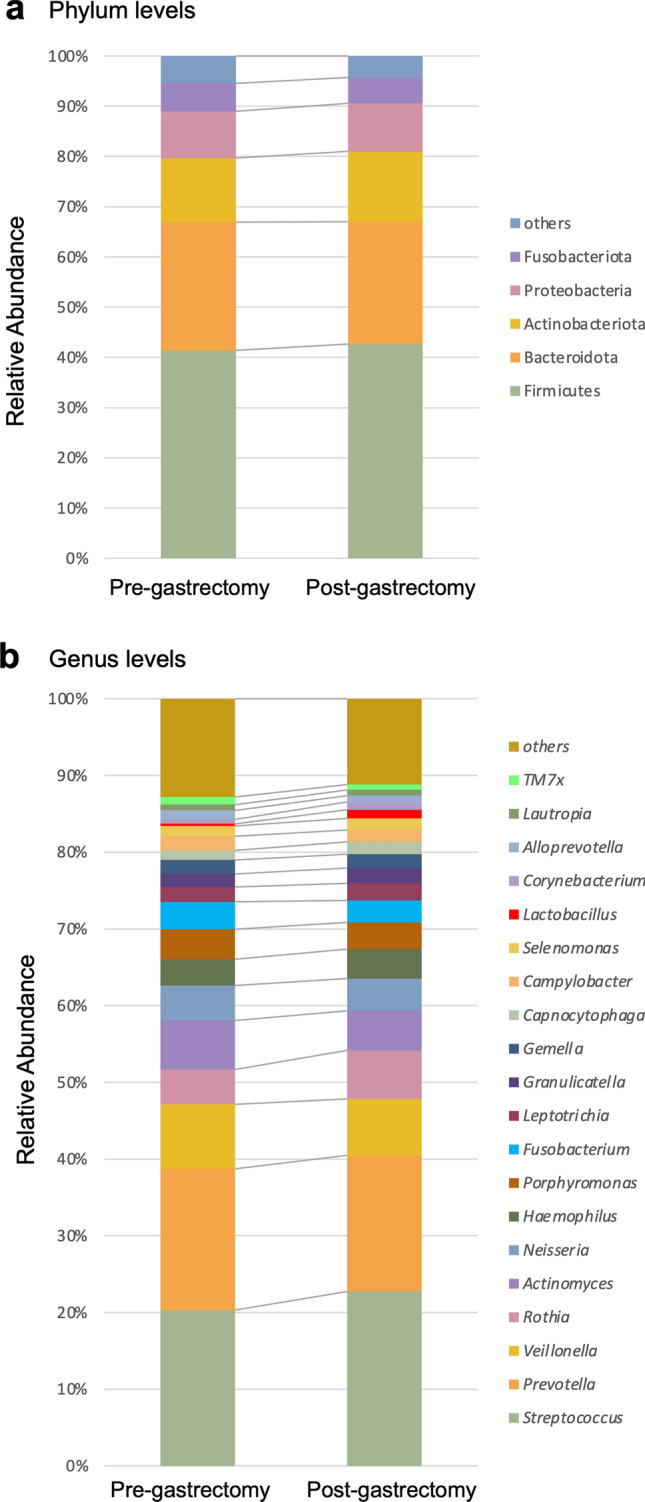
Taxonomic composition of salivary microbiota. Vertical bar plot showing the relative abundance of bacterial phyla (**a**) and genera (**b**) at the time points of pre- and post-gastrectomy. (**a**) Firmicutes, Bacteroidota, Actinobacteriota, Proteobacteria, and Fusobacteriota were the five most abundant phyla in all subjects, comprising 94.55 and 95.73% of the total bacterial communities at the time point of pre- and post-gastrectomy, respectively. (**b**) Bar plot of the 20 most abundant genera at the time point of pre- and post-gastrectomy.

Salivary bacteria with a relative abundance of at least 0.1% in pre- and post-gastrectomy groups were classified into 11 phyla, 17 classes, 38 orders, 62 families, and 112 genera. The predominant bacteria (> 5% of total sequences in either group) at the phylum level were Firmicutes and Bacteroidota, followed by Actinobacteriota, Proteobacteria, and Fusobacteriota, in total, accounting for 94.55 and 95.73% of the salivary microbiota in the pre- and post-gastrectomy groups, respectively (Fig. 1a). At the genus level, the pre-gastrectomy group included 269 genera, out of which 91 were absent in the post-gastrectomy group. In contrast, the post-gastrectomy group was characterized by 204 genera, of which 26 were absent from the pre-gastrectomy group. That is, the pre- and post-gastrectomy salivary microbiota shared about 60.3% of the bacterial genera; 30.8% were found only pre-gastrectomy and 8.8% only post-gastrectomy. A total of 62 genera were found in at least 50% of the subjects in both groups, with 53 being shared by both. The 20 most abundant genera in the pre- and post-gastrectomy groups accounted for 87.19 and 88.82% of the total genera abundance in the two groups, respectively (Fig. 1b).

### Changes in the number of bacterial species pre- and post-gastrectomy

**Figure 2:**
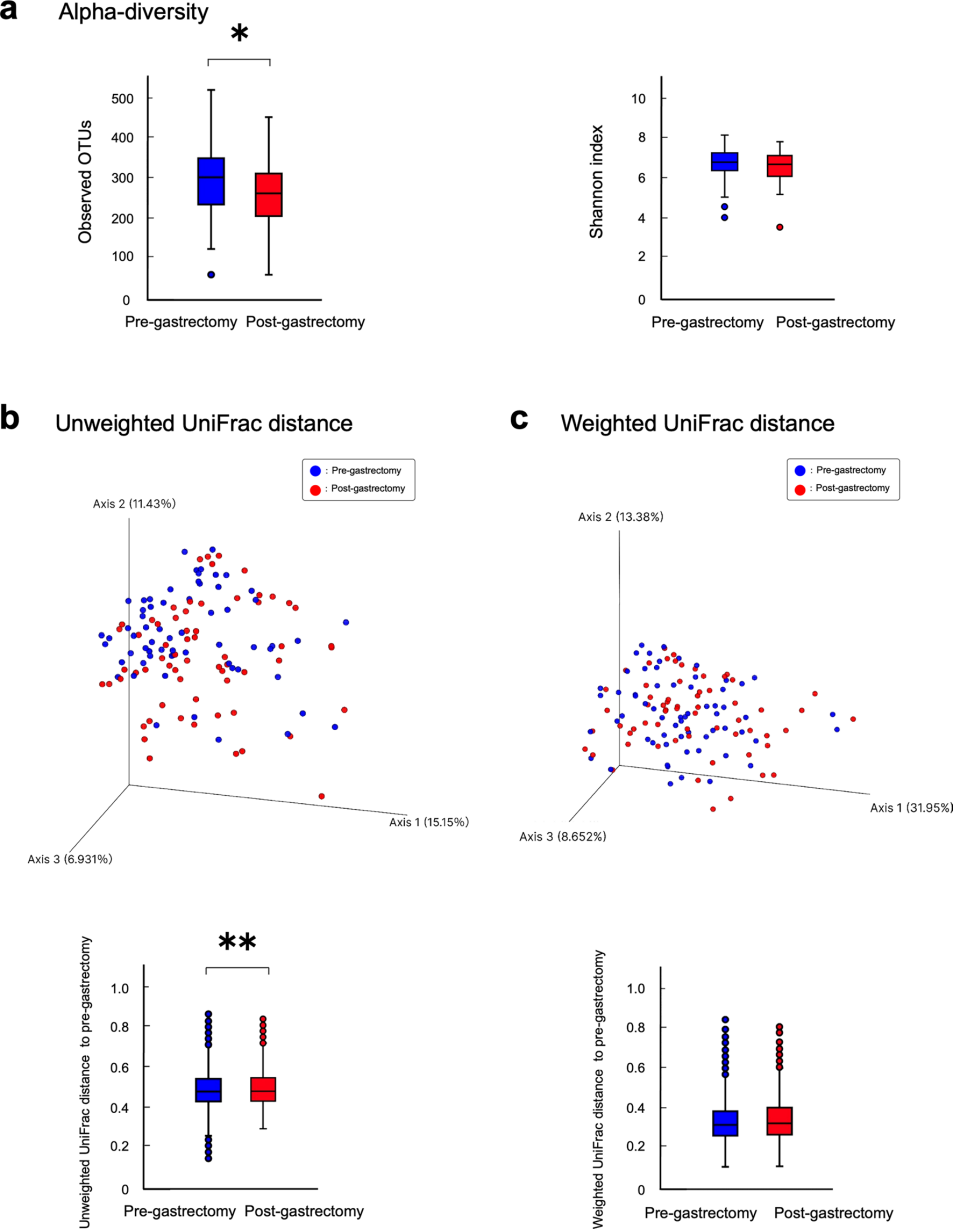
Alpha and beta diversity of salivary microbiota. (**a**) Alpha diversity, observed operational taxonomic unit (OTU) index, and Shannon index of 63 patients at the time point of post-gastrectomy (red) compared to pre-gastrectomy (blue). *, *p* < 0.05. Comparisons between groups were performed using the Kruskal -Wallis test. (**b**) Beta diversity; Unweighted UniFrac distances. (**c**) Beta diversity; Weighted UniFrac distances. Principal coordinate analysis (PCoA) plots of samples from 63 patients at the time point of pre-gastrectomy (blue) and post-gastrectomy (red). Box plots represent UniFrac distances at the time point of post-gastrectomy (red columns) from pre-gastrectomy (blue columns). **, *p* < 0.01. Comparisons between groups were performed using PERMANOVA, 999 permutations.

In the salivary microbiota, analysis of alpha diversity showed that the species richness of bacteria decreased significantly in the post-gastrectomy group compared with those in the pre-gastrectomy group (observed operational taxonomic unit (OTU) index, *p* = 0.018; Fig. 2a), while there was no significant difference in the evenness of oral bacteria (Shannon index, *p* = 0.147). In contrast, in the gastric fluid microbiota, the observed OTU and Shannon index revealed significantly different pre- and post-gastrectomy groups (observed OTU index, *p* = 0.012; Shannon index,* p* = 0.027; Fig. S1a), indicating a decrease in the species richness and the evenness of gastric bacteria, which is consistent with our previous report^25^. In addition, the number of bacterial species shared by the same individual’s saliva and gastric fluid pairs was approximately 9.7% lower post-gastrectomy than pre-gastrectomy (*p* < 0.001; Fig. S2).

### Change in microbial structure pre- and post-gastrectomy

According to the Principal Coordinate Analysis (PCoA) plots based on the unweighted UniFrac distance metric, the overall structure of the pre-and post-gastrectomy groups' salivary microbiota was distinguished in a 3-dimensional space. This compositional difference between the two groups was verified by PERMANOVA based on the unweighted UniFrac data (999 permutations, *p* = 0.003; Fig. 2b). No statistically significant differences were observed in the microbial structure among the two groups using the weighted UniFrac distance metric (Fig. 2c). Consistent with our previous report^25^, unweighted UniFrac and weighted UniFrac distance revealed that the gastric microbiota was significantly different between the pre- and post-gastrectomy groups (Unweighted UniFrac distance, *p* = 0.013, Weighted UniFrac distance, *p* = 0.001; Fig. S1b, c).

The differences in the microbial structure of saliva and gastric fluid were confirmed at each time point of pre- and post-gastrectomy (Fig. S3). The microbial structure of saliva and gastric fluid did not differ significantly between the pre- and post-gastrectomy periods (Fig. S4). The change in microbial structure pre- and post-gastrectomy was markedly more significant in gastric fluid than in saliva (Weighted UniFrac distance, *p* < 0.001; Fig. S5).

### Bacteria in saliva and gastric fluid with different abundances pre- and post-gastrectomy

**Figure 3:**
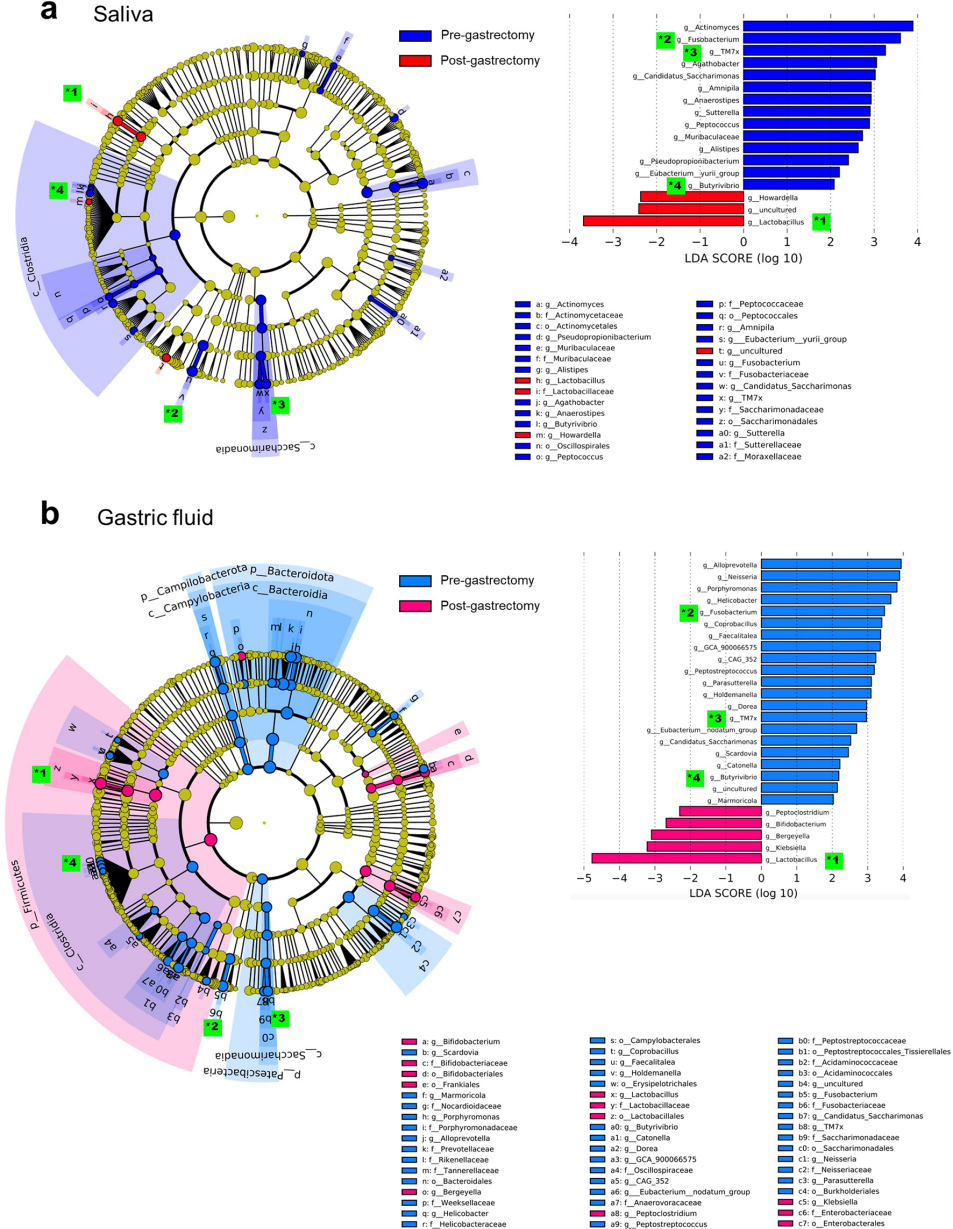
The differentially abundant bacterial genera between pre- and post-gastrectomy groups identified by linear discriminant analysis effect size (LEfSe). The Salivary microbiota (**a**) and gastric microbiota (**b**) are shown. Cladogram of differentially abundant bacterial taxa with each layer representing a different taxonomy. The enriched taxa in pre-gastrectomy (blue or light blue) or post-gastrectomy (red or pink) groups of microbiotas are represented in the cladogram. The central point represents the root of the tree (bacteria), and each ring represents the next lower taxonomic level (phylum to genus; p, phylum; c, class; o, order; f, family; g, genus). The diameter of each circle represents the relative abundance of the taxon. Histogram of the linear discriminant analysis (LDA) scores for differentially abundant bacterial taxa between the pre- and post-gastrectomy groups. LDA scores ≥ 2.0 are shown. The red or pink color represents significantly increased taxa in the post-gastrectomy group than in the pre-gastrectomy group. The blue or light blue color represents significantly abundant taxa in the pre-gastrectomy group. Bacteria found in common in the salivary and gastric microbiota at the genus level are marked with *. 1*, *Lactobacillus*; 2*, *Fusobacterium*; 3*, *TM7x*; and 4*, *Butyrivibrio*.

Bacteria with different abundances pre- and post-gastrectomy were identified by linear discriminant analysis effect size (LEfSe) analysis in saliva and gastric fluid. The cladogram in Fig. 3 represents the taxa that differed significantly between the pre- and post-gastrectomy groups in a taxonomic hierarchy from phylum to genus.

At the genus levels, the salivary microbiota showed an increased abundance of *Lactobacillus* and *Howardella* and a decreased abundance of *Actinomyces*, *Fusobacterium*, *TM7x*, and *Butyrivibrio*, among others in the post-gastrectomy group compared to the pre-gastrectomy group (Fig. 3a). The gastric fluid microbiota showed a higher abundance of *Lactobacillus* and *Klebsiella*, among others, while displaying a lower abundance of *Alloprevotella*, *Neisseria*, *Porphyromonas*, *Helicobacter*, *Fusobacterium*, *TM7x*, and *Butyrivibrio*, among others in the post-gastrectomy group compared to the pre-gastrectomy group (Fig. 3b), consistent with our previous report^25^. These results indicate that the abundance of bacteria at the genus level significantly differed in gastric fluid and saliva pre- and post-gastrectomy.

### Bacteria common to saliva and gastric fluid with changed abundance pre- and post-gastrectomy

By comparing the results of LEfSe in the saliva and gastric fluid, we found some bacterial genera that showed a marked increase or decrease in the salivary microbiota in the post-gastrectomy group compared to the pre-gastrectomy group showing a concomitant increase or decrease in the gastric fluid microbiota. For example, the abundance of *Lactobacillus* in the salivary microbiota of the post-gastrectomy group was increased compared to the pre-gastrectomy group, and a similar increase was observed in the gastric fluid microbiota. Similarly, the abundance of *Fusobacterium*, *TM7x*, and *Butyrivibrio* in the salivary microbiota of the post-gastrectomy group was decreased, and a similar decrease was observed in the gastric fluid microbiota (Fig. 3).

**Figure 4:**
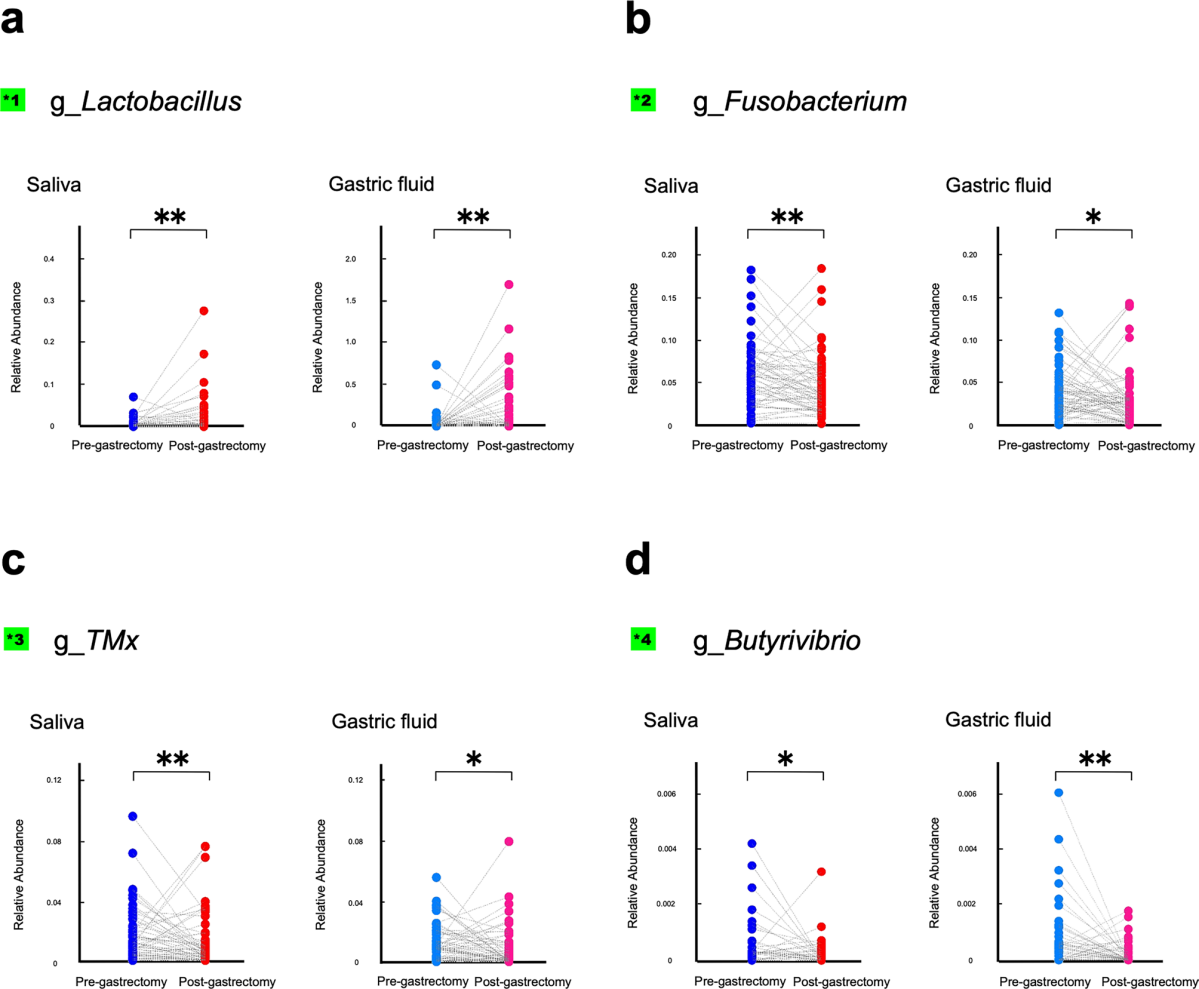
Changes in the relative abundance of salivary and gastric bacteria. Relative abundance of salivary and gastric bacteria at the time points pre- and post-gastrectomy. (**a**) *Lactobacillus*, (**b**) *Fusobacterium*, (**c**) *TM7x*, and (**d**) *Butyrivibrio*. **, *p* < 0.01; *, *p* < 0.05. Comparisons between the groups were performed using the Wilcoxon signed-rank sum test.

Therefore, we compared the pre- and post-gastrectomy relative amounts of these four individual bacterial genera in pairs. The results demonstrated that *Lactobacillus* in the salivary microbiota and gastric fluid microbiota in the post-gastrectomy group was significantly higher than that of the pre-gastrectomy group *(p* < 0.01), while *Fusobacterium*, *TM7x*, and *Butyrivibrio* were substantially lower (*p* < 0.05; Fig. 4).

## Discussion

In the present study, we have characterized the changes in the salivary microbiota composition associated with the resection of gastric cancer using 16S rRNA metagenomic analysis. The number of bacterial species in the salivary microbiota decreased, and the bacterial composition changed after the resection of gastric cancer. In addition, we identified several bacterial genera that varied significantly in the salivary microbiota, some of which also show similar changes in the gastric microbiota. These data reveal that changes in the gastric environment affect the oral microbiota, emphasizing the close association between the oral and gastric fluid microbiota. Our study highlights the importance of focusing on the oral microbiota in the perioperative period of gastrectomy in patients with gastric cancer.

There is increasing evidence that the oral microbiota is closely associated with systemic conditions^27^. Changes in systemic status may affect the oral microbiota, with reports based on data from animal studies, and a few studies have been conducted on humans^28^. In this study, the changes in the gastric environment due to gastric cancer resection may have affected the systemic metabolic status, which in turn may have affected the oral microbiota. Gastrectomy causes significant physiological and anatomical changes in the gastrointestinal tract, altering conditions such as pH, oxygenation, food transit time, intestinal motility, and hormonal status, affecting the composition of the intestinal microbiota and leading to an impact on the systemic status^29^. Case studies of gastrectomy treatment for morbid obesity have reported changes not only in the gut microbiota and metabolome but also in the oral microbiota^30^. However, the mechanisms behind gut and oral microbiota composition changes observed after gastrectomy are not fully understood, and further animal studies are needed to elucidate them. Another factor that may affect oral microbiota composition post-gastrectomy is gastroesophageal reflux. Gastrectomy increases the risk of gastroesophageal reflux and may affect oral pH. A known postoperative complication of gastrectomy is reflux esophagitis, caused by the reflux of gastric contents, including stomach acid, into the oral cavity. It has been reported that the oral microbiota of patients with reflux esophagitis differs from that of healthy individuals^31,32^. Therefore, changes in the oral microbiota after gastric cancer resection may be due to gastroesophageal reflux. In this study, about 60.3% of the salivary bacterial genera were commonly present pre- and post-gastrectomy, about 30.8% were found only pre-gastrectomy, and about 8.8% were found only post-gastrectomy. Factors leading to such significant changes are the subject of future research.

Recently, it has become clear that more bacteria than previously thought are transferred from the mouth to the intestine in healthy individuals^33^. One in three salivary bacteria species pass through the digestive tract, colonize the gut, and make up at least 2% of the intestinal microbiota^34^. However, little is known about oral bacteria in the stomach. Some reports show that oral bacteria are abundant in the stomachs of gastric cancer patients^35,36^. The results of this study showed that the number of bacterial genera shared between saliva and gastric fluids of the same person decreased by about 9.7% post-gastrectomy compared to pre-gastrectomy (Fig. S2); moreover, the abundance of some bacteria commonly change in saliva and gastric fluid before and after surgery, signifying a close relationship between the oral and gastric microbiota. Although the microbiota of gastric fluid and gastric mucosa differ, both are associated with gastric cancer and may be important in maintaining gastric homeostasis^37–39^. Our findings suggest that more research into the details of oral bacteria transfer into the stomach is needed. Future evaluation of the microbiota of gastric cancer tissues will further clarify the relationship between the oral microbiota and the bacteria colonizing the gastric mucosa.

In the present study, the abundance of certain oral bacteria present in saliva and gastric fluid was significantly altered by resection of gastric cancer. Among them, the abundance of *Lactobacillus* increased, while that of *Fusobacterium*, *TM7x*, and *Butyrivibrio* decreased in both saliva and gastric fluid post-gastrectomy. *Lactobacillus* is a genus of bacteria that includes *Lactobacillus casei,* involved in the re-establishment of pH homeostasis in the oral cavity^40^. Conversely, it should be noted that *Lactobacillus* is frequently observed in the stomach of patients with gastric cancer, and its abundance increases after surgery^41^. Furthermore, it is a genus of bacteria attracting attention for its function as a probiotic. *Fusobacterium* is more abundant in gastric cancer patients' oral cavities and stomachs than in healthy individuals^42^. Interestingly, the reduction in the abundance of this genus after resection of gastric cancer may have approached normality. Since an increase in *Lactobacillus* and a decrease in *Fusobacterium* in gastric fluid is seen under immunosuppression^43^, similar changes seen in the postoperative period may be significant. *Fusobacterium* and *TM7* are also known to be abundant in the gastric mucosa of gastric cancer patients^44^. Members of *TM7* are associated with the promotion of inflammation, including periodontal disease and inflammatory bowel disease. *TM7x* has been reported to establish a stable long-term association with several *Actinomyces* species^45^. In this research, we found that *Actinomyces* in oral microbiota decrease with *TM7x* in the post-gastrectomy group, suggesting that *TM7x* could be in a parasitic relationship with gastric *Actinomyces*. Interestingly, these bacterial groups associated with gastric cancer were reduced in both saliva and gastric fluid after surgery. The functions of individual bacteria need to be further elucidated in a separate study.

Recent reports have shown that periodontal disease is a risk factor for infectious complications after gastrointestinal surgery^23^, indicating the importance of perioperative oral hygiene. The findings of this study that the oral microbiota changes before and after surgery and that it is also associated with gastric fluid microbiota suggest that appropriate oral intervention may be critical. Understanding the symbiotic relationship between the oral and gastric microbiota and the host and the nature of the symbiotic bacteria will make this possible in the future.

The 16S rRNA metagenomic analysis technique used in this study does not determine whether the bacteria in the sample are alive or dead in vivo. Other methods are needed to distinguish between viable and dead bacteria, such as conventional bacterial culture, direct viable count and the double-staining method using epifluorescence microscopy, inhibitory substance-influenced molecular methods, and flow cytometry^46^. Additionally, if sufficient sequencing depth can be obtained, there are methods to evaluate bacterial viability from sequencing data sets^47,48^. Since the purpose of this study was the preliminary evaluation of the oral and gastric microbiota before and after surgery, both viable and dead bacteria were assessed. Furthermore, this cross-sectional study observed a phenomenon; therefore, causality is unknown and all confounding factors cannot be eliminated. As for smoking status, we performed an analysis to exclude this factor. Smoking and smoking cessation are known to alter the oral microbiota^49,50^. Thirteen current smokers were included in the study; they had temporarily stopped smoking one month prior to surgery and postoperative smoking status was not confirmed. Even after excluding these subjects, this study's conclusions remained consistent: the salivary microbiota changed pre- and post-gastrectomy, and there was a common group of bacteria that changed in saliva and gastric fluid (Fig. S6). The trend of pre- and post-gastrectomy changes in certain bacterial genera did not vary when smokers were excluded, but there were some bacterial genera for which the differences were no longer significant (Fig. S7). These results suggest that future study designs should take into account smoking and smoking cessation factors in larger systems. Evaluation in animal studies is required to clarify other factors. These are some of the limitations of this study.

In conclusion, we identified changes in the composition of the salivary microbiota in patients undergoing gastric cancer resection and discovered an association between changes in the salivary and gastric fluid microbiota. The current findings may provide an essential foundation for developing novel therapies that control the oral microbiota of gastric cancer patients, thereby leading to a favorable prognosis.

## Methods

### Participants

This study was conducted in accordance with the Helsinki Declaration and its latest amendments. It was approved by the Ethics Committee of Osaka Medical and Pharmaceutical University, Takatsuki City, Japan (approval no. 2145). Furthermore, written informed consent was obtained from all participants.

The study participants included 63 patients diagnosed with primary gastric cancer. They underwent distal gastrectomy and B1 or RY reconstruction according to the Japanese Guidelines for the Treatment of Gastric Cancer^51^, at the Department of General and Gastroenterological Surgery, Osaka Medical and Pharmaceutical University Hospital, Takatsuki, Japan, between January 2019 and February 2021. The exclusion criteria were as follows: patients who had macroscopic residual disease at the time of surgery (R2 resection); patients who underwent neoadjuvant chemotherapy; those that underwent simultaneous resection of other organs for malignant illnesses; those that had pyloric stenosis, and those on immunosuppressive drugs, corticosteroids, and acid-suppressing drugs, or antibiotics three months preceding the sample collection; and those currently on treatment for infectious diseases, autoimmune disease, renal or liver failure, or malignant tumors. The characteristics, cancer classification, and surgical approach of the participants are summarized in Table 1.

**Table 1:** Characteristics of the study population (n = 63).

General conditions	
Age (years)	71.8 ± 9.6
Sex (M/F)	42/21
Body mass index (kg/m^2^): pre-/post-gastrectomy (mean ± SD)	22.5 ± 3.0/20.4 ± 3.1
Never smoker/Ex-smoker/Current smoker	29/21/13
Dyslipidemia^a^	18 (28.6%)
Hypertension^b^	32 (50.8%)
Diabetes^c^	9 (14.3%)
Gastric cancer status	
Clinical stage: (I/II/III/IV)	47/6/9/1
Surgical approach (Laparoscopic/open/robotic)	53/3/7
Reconstructive methods after gastric cancer resection (B1/RY)	37/26
Oral conditions	
Number of teeth (median [min–max])	22.5 [0–28]
Denture wearing	29 (46.9%)
Severe periodontitis^d^	13 (19.4%)
Number of teeth treated with caries (median [min–max])	9.5 [0–28]

^a^Dyslipidemia is defined as LDL-C > 140 mg/dl, HDL-C < 40 mg/dl, and triglycerides > 150 mg/dl or the use of anti-dyslipidemic drugs.

^b^Hypertension is defined as blood pressure > 140/90 mmHg or the use of anti-hypertensive drug.

^c^Diabetes is defined as HbA1c > 6.5% (NGSP) or the use of oral anti-diabetic drugs or insulin therapy. B1; Billroth 1, RY; Roux-en-Y.

^d^Criteria for staging according to the World Workshop on the Classification of Periodontal and Peri-implant Diseases and Conditions^52^.

The patients in this study were referred to the Department of Dental and Oral Surgery at Osaka Medical and Pharmaceutical University for perioperative oral management when surgery was scheduled. Specialized dentists examined the patients’ oral conditions before and after surgery and provided professional oral management. Oral information, such as number of remaining teeth, denture wear status, periodontal disease status, and caries status, is summarized in Table 1. No significant changes in oral status were observed before and after surgery.

### Sample collection

Saliva and gastric fluid samples were collected from the same 63 patients at two time points, before and six months after gastrectomy. Some of the gastric fluid samples used in this study were part of our previously reported sample^25^, and their bacterial sequence information has already been registered in DDBJ https://www.ncbi.nlm.nih.gov/bioproject/PRJDB12280.

Saliva and gastric fluid samples were collected as previously described^25,53^, with some modifications. Briefly, after fasting overnight and before eating, a saliva sample was collected with the cotton swab method using the saliva collection system SalivaBio® Oral Swab and Swab Storage Tube (Salimetrics, State College, PA, USA). After that, the gastric sample was collected during upper gastrointestinal endoscopy by aspirating as much gastric fluid as possible with a disposable syringe connected to a sterile tube. Immediately after collection, the samples were frozen and stored at − 80 °C until the DNA was extracted.

### DNA extraction, 16S rRNA sequencing, and taxonomic classification

DNA extraction, 16S rRNA sequencing, and taxonomic classification were performed using previously reported methods^53^. Briefly, 30 μL of saliva or gastric fluid samples were homogenized by the bead method, and bacterial genomic DNA was extracted using GENE PREP STAR PI-480 (Kurabo Industries Ltd., Osaka, Japan). The V1-V2 region of the 16S rRNA gene was amplified using primer set 27Fmod and 338R, and libraries were prepared according to the 16S Metagenomics Sequencing Library Preparation Protocol (Illumina, CA, San Diego, USA). DNA sequencing was performed using the MiSeq Reagent Kit v2 (Illumina) for 500 cycles. An average of 38,068 sequence reads of 250 bp paired-end reads were combined with forward and reverse FASTQ reads using QIIME2 (Quantitative Insights into Microbial Ecology 2) version 2022.08, quality filtered, and noise elimination was performed. After quality filtering, 3,972,313 sequences were obtained, averaging 30,225 sequences per sample (min: 16,629, max: 53,802). A rarefaction minimum depth cutoff of 10,000 was selected as the total sample for the downstream analysis. These were clustered into operational taxonomic units (OTUs) according to a 97% similarity cutoff and assigned to the curated Silva 138 reference database.

### Statistical analysis

The within-subject alpha diversity of bacterial communities was evaluated using the observed OTU index and Shannon index. The Kruskal–Wallis test was used for comparisons between the groups, and statistical significance was set at *p* < 0.05. Beta diversity between subjects was evaluated using unweighted and weighted UniFrac distance metrics. To visualize the global differences in the microbiota structure in the UniFrac analysis, we performed Principal Coordinate Analysis (PCoA). The significance of compositional differences between groups was assessed by permutational multivariate analysis of variance (PERMANOVA). QIIME2 was used for these analyses.

The linear discriminant analysis effect size (LEfSe) algorithm was used to detect differentially abundant bacteria between the two groups. All analyses were run with LEfSe's α parameter for pairwise tests set at 0.05, and the threshold of the logarithmic score for LDA analysis was set at 2.0.

The relative abundance of specific bacterial genera was compared in pairs between pre- and post-gastrectomy groups using the Wilcoxon signed-rank sum test, and statistical significance was set at* p* < 0.05. The statistical software JMP Pro 15.1.0 (SAS Institute, Inc., Cary, NC, USA) was used for these analyses.

### Ethical approval

All procedures conducted in studies involving human participants were performed in accordance with the ethical standards of the Ethics Committee of Osaka Medical College, approval no. 2145, Chugoku Central Hospital of the Mutual Aid Association of Public School Teachers, approval no. 1905-02, with the 1964 Helsinki Declaration and its later amendments or comparable ethical standards.

### Supplementary Information

Supplementary Information.

## Data Availability

The datasets used and/or analyzed in this study are available upon reasonable request. If someone wants to request the data from this study, he/she may request them from the corresponding author.
