# Supplementary material for: Changes in salivary microbiota due to gastric cancer resection and its relation to gastric fluid microbiota
Source: Sci Rep. 2023 Sep 22;13:15863. doi: 10.1038/s41598-023-43108-8 (PMC10516953; doi:10.1038/s41598-023-43108-8)
Supplement: Supplementary file 1 — Supplementary Information. [file 41598_2023_43108_MOESM1_ESM.pdf]

Supplementary Information

Changes in salivary microbiota due to gastric cancer resection and its relation to gastric fluid microbiota

Eri Komori, Nahoko Kato-Kogoe, Yoshiro Imai, Shoichi Sakaguchi, Kohei Taniguchi, Michi Omori, Mayu Ohmichi, Shota Nakamura, Takashi Nakano, Sang-Woong Lee, and Takaaki Ueno

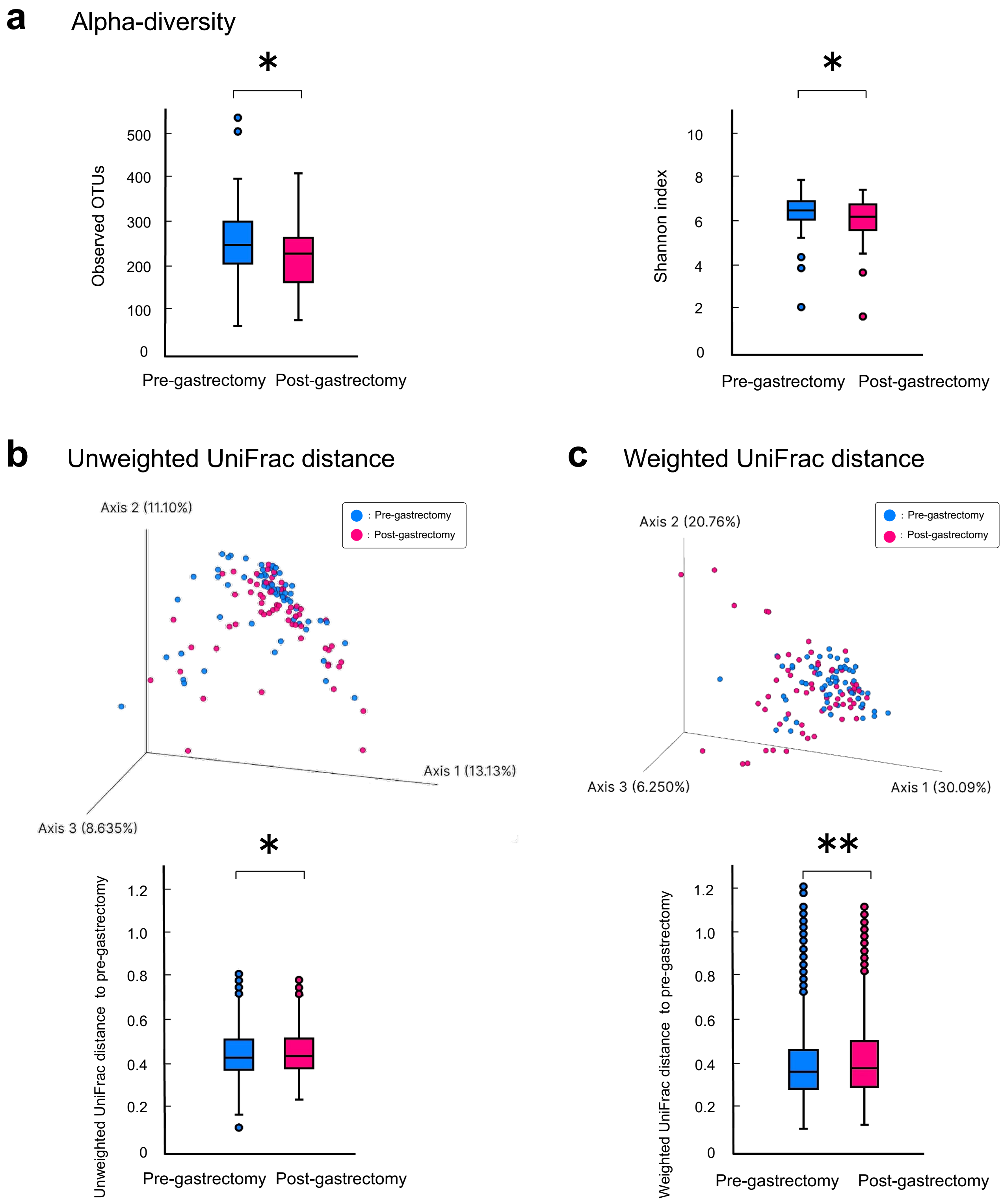

Fig. S1. Alpha and beta diversity of gastric microbiota at the time of pre- and post-gastrectomy.

(a) Alpha diversity, observed operational taxonomic unit (OTU) index, and Shannon index of 63 patients at the time point of post-gastrectomy (pink) compared to pre-gastrectomy (light blue). *, $p < 0.05$. Comparisons between groups were performed using Kruskal -Wallis test.

(b) Beta diversity; Unweighted UniFrac distances.

(c) Beta diversity; Weighted UniFrac distances.

Principal coordinate analysis (PCoA) plots of samples from 63 patients at the time points pre-gastrectomy (light blue) and post-gastrectomy (pink). Box plots represent UniFrac distances at the time points post-gastrectomy (pink columns) and pre-gastrectomy (light blue columns). *, $p < 0.05$. **, $p < 0.01$. Comparisons between groups were performed using PERMANOVA, 999 permutations.

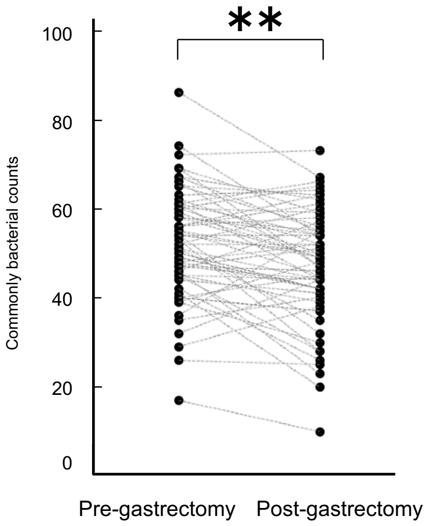

Fig. S2. Change of the number of bacterial species commonly present in the saliva and the gastric fluid.

The number of bacterial species commonly present in the saliva and the gastric fluid at the time of pre- and post-gastrectomy in the same individual are shown. **, $p < 0.01$. Comparisons between the pre- and post-gastrectomy groups were performed using Wilcoxon signed-rank sum test.

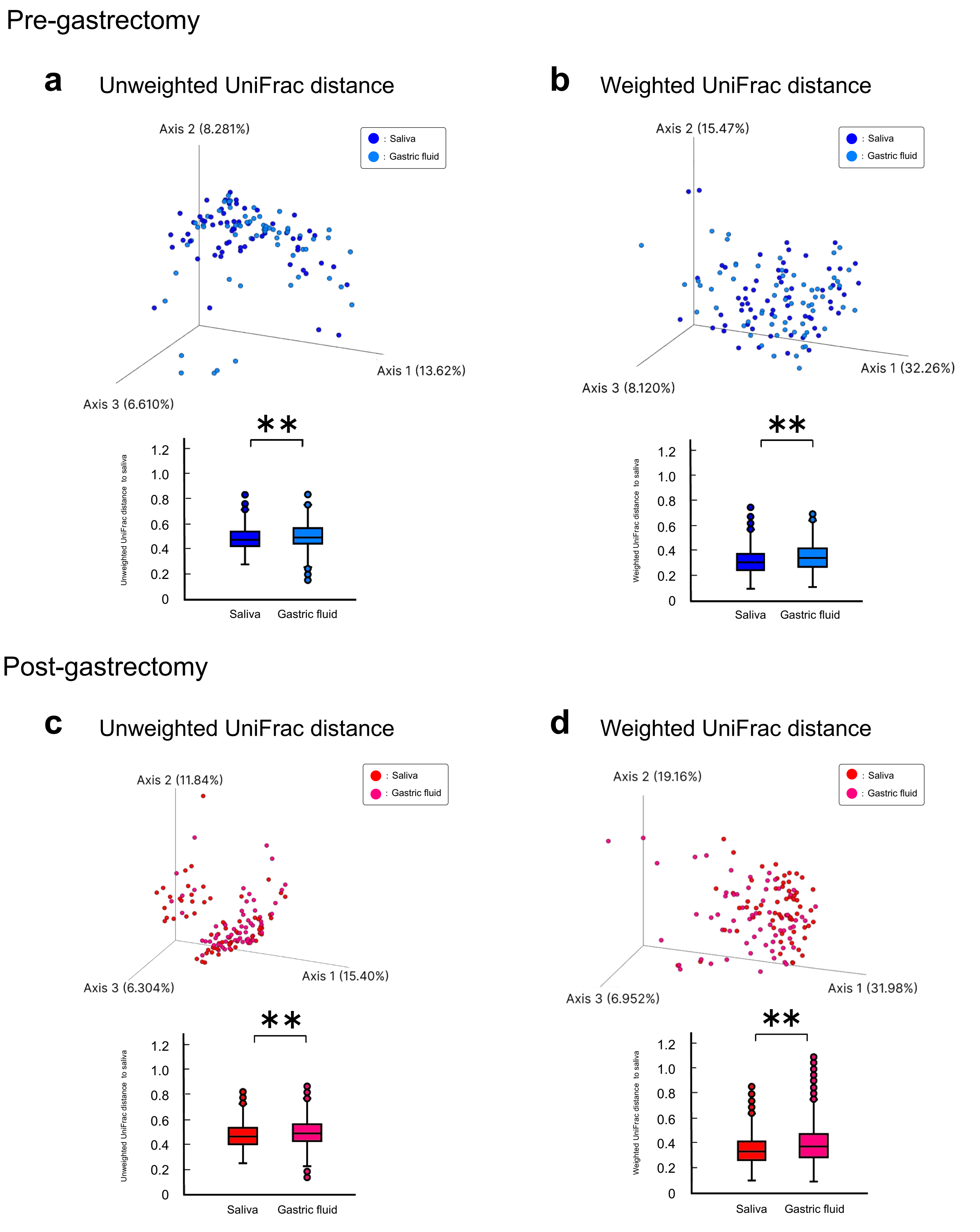

Fig. S3. Beta diversity of saliva and gastric fluid microbiota.
Unweighted UniFrac distances and Weighted UniFrac distances of saliva and gastric fluid at the time point of pre-gastrectomy (**a** and **b**) and post-gastrectomy (**c** and **d**) are shown. Principal coordinate analysis (PCoA) plots of samples from 63 patients at the time point of pre-gastrectomy (saliva; blue, and gastric fluid; light blue) and post-gastrectomy (saliva; red, and gastric fluid; pink). Box plots represent UniFrac distances saliva from gastric fluid. **, $p < 0.01$. Comparisons between groups were performed using PERMANOVA, 999 permutations.

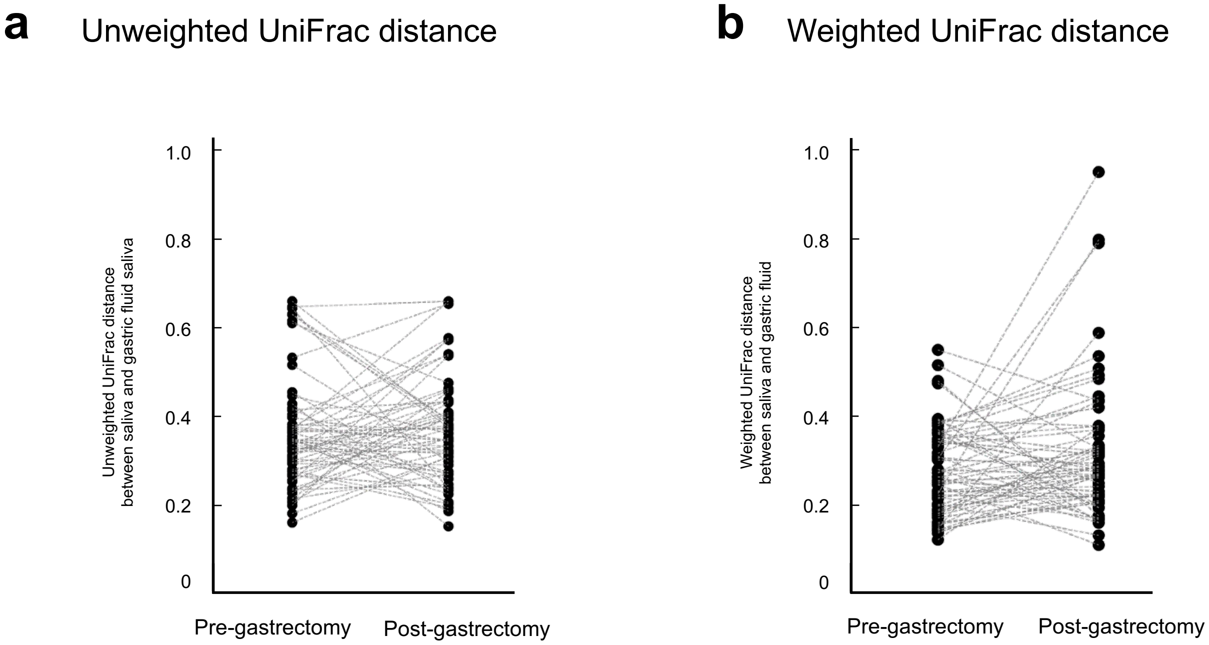

Fig. S4. The differences between saliva and gastric fluid microbiota at the time of pre- and post-gastrectomy.

Unweighted UniFrac distances **(a)** and Weighted UniFrac distances **(b)** between saliva and gastric fluid in the same individual are shown. Comparisons between the pre- and post-gastrectomy groups were performed using Wilcoxon signed-rank sum test and no significant difference.

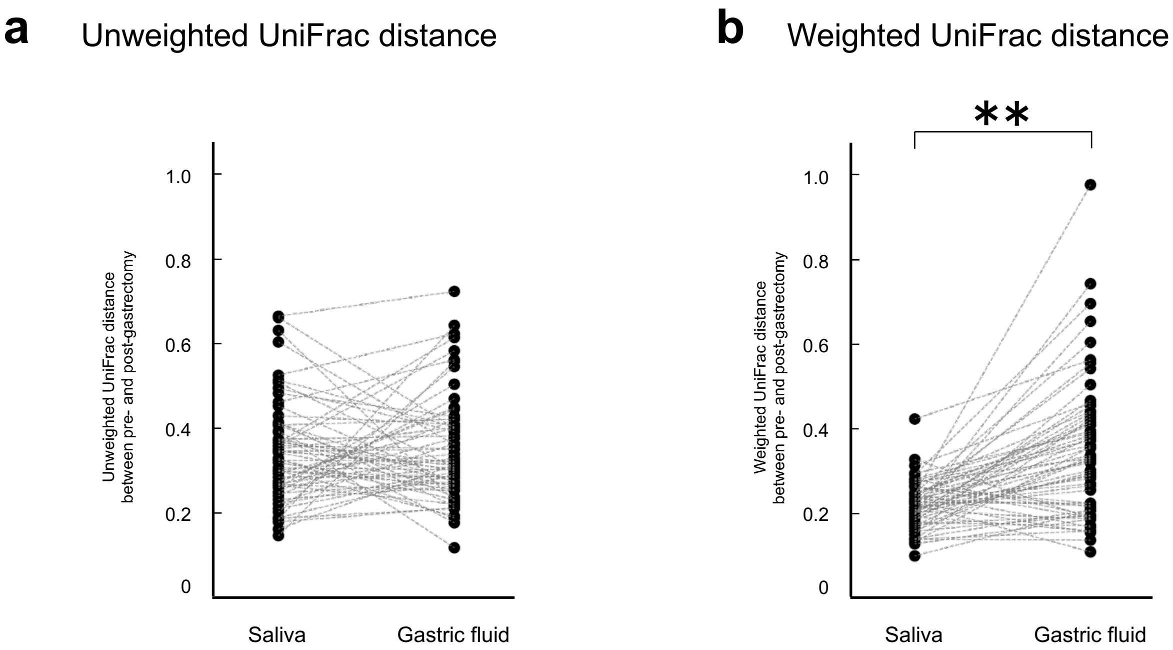

Fig. S5. The change in microbial structure pre- and post-gastrectomy.
Unweighted UniFrac distances (**a**) and Weighted UniFrac distances (**b**) between pre- and post-gastrectomy in the same individual are shown. **, $p < 0.01$. Comparisons between the saliva and gastric fluid groups were performed using Wilcoxon signed-rank sum test.

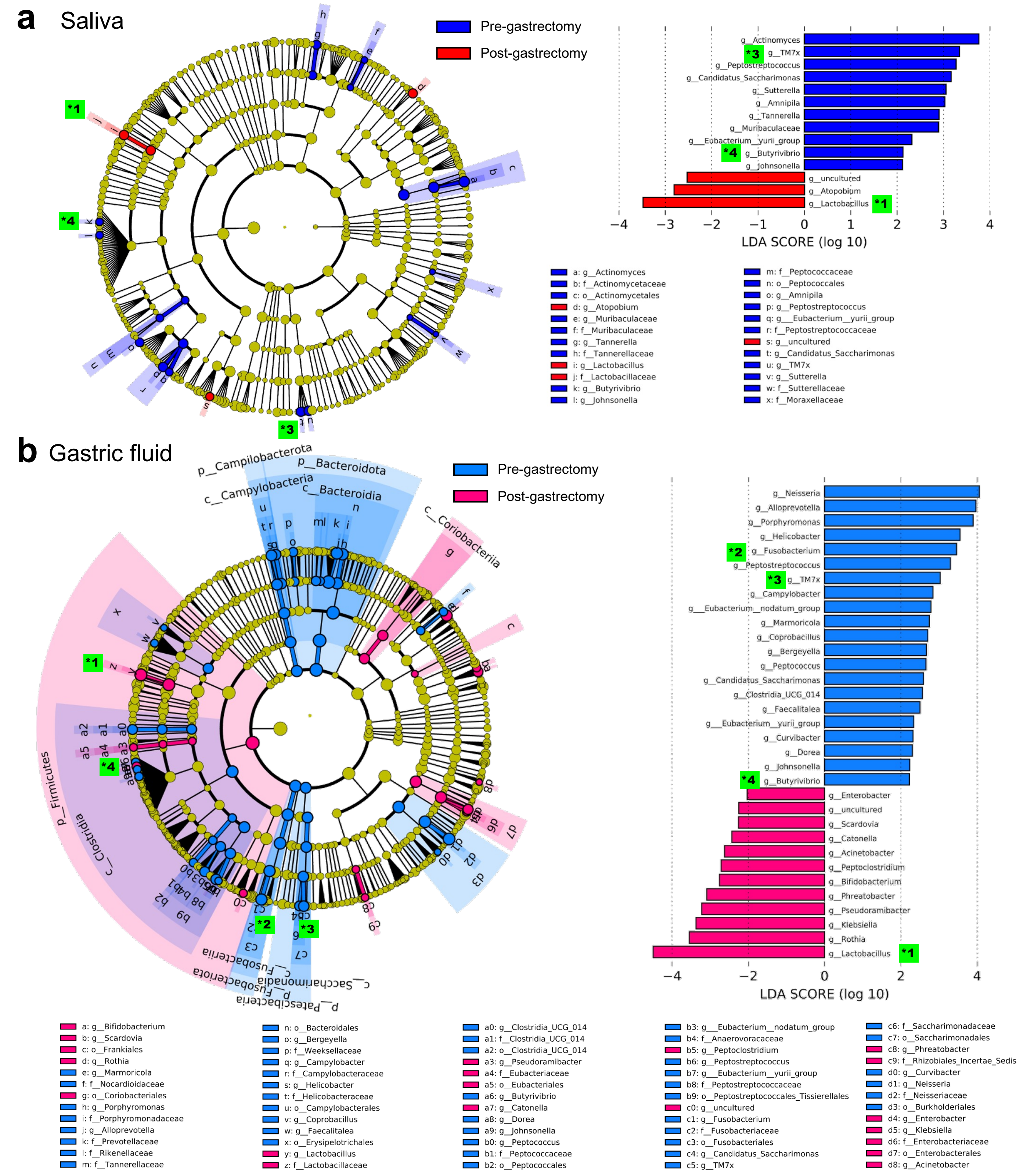

Fig. S6. The differentially abundant bacterial genera between pre- and post-gastrectomy groups identified by linear discriminant analysis effect size (LEfSe) with the exclusion of 13 current smokers from the results in Figure 3.

The Salivary microbiota (**a**) and gastric microbiota (**b**) are shown. Cladogram of differentially abundant bacterial taxa with each layer representing a different taxonomy. The enriched taxa in pre-gastrectomy (blue or light blue) or post-gastrectomy (red or pink) groups of microbiotas are represented in the cladogram. The central point represents the root of the tree (bacteria), and each ring represents the next lower taxonomic level (phylum to genus; p, phylum; c, class; o, order; f, family; g, genus). The diameter of each circle represents the relative abundance of the taxon. Histogram of the linear discriminant analysis (LDA) scores for differentially abundant bacterial taxa between the pre- and post-gastrectomy groups. LDA scores ≥ 2.0 are shown. The red or pink color represents significantly increased taxa in the post-gastrectomy group than in the pre-gastrectomy group. The blue or light blue color represents significantly abundant taxa in the pre-gastrectomy group. Bacteria found in common in the salivary and gastric microbiota at the genus level are marked with *. 1*, *Lactobacillus*; 2*, *Fusobacterium*; 3*, *TM7x*; and 4*, *Butyrivibrio*.

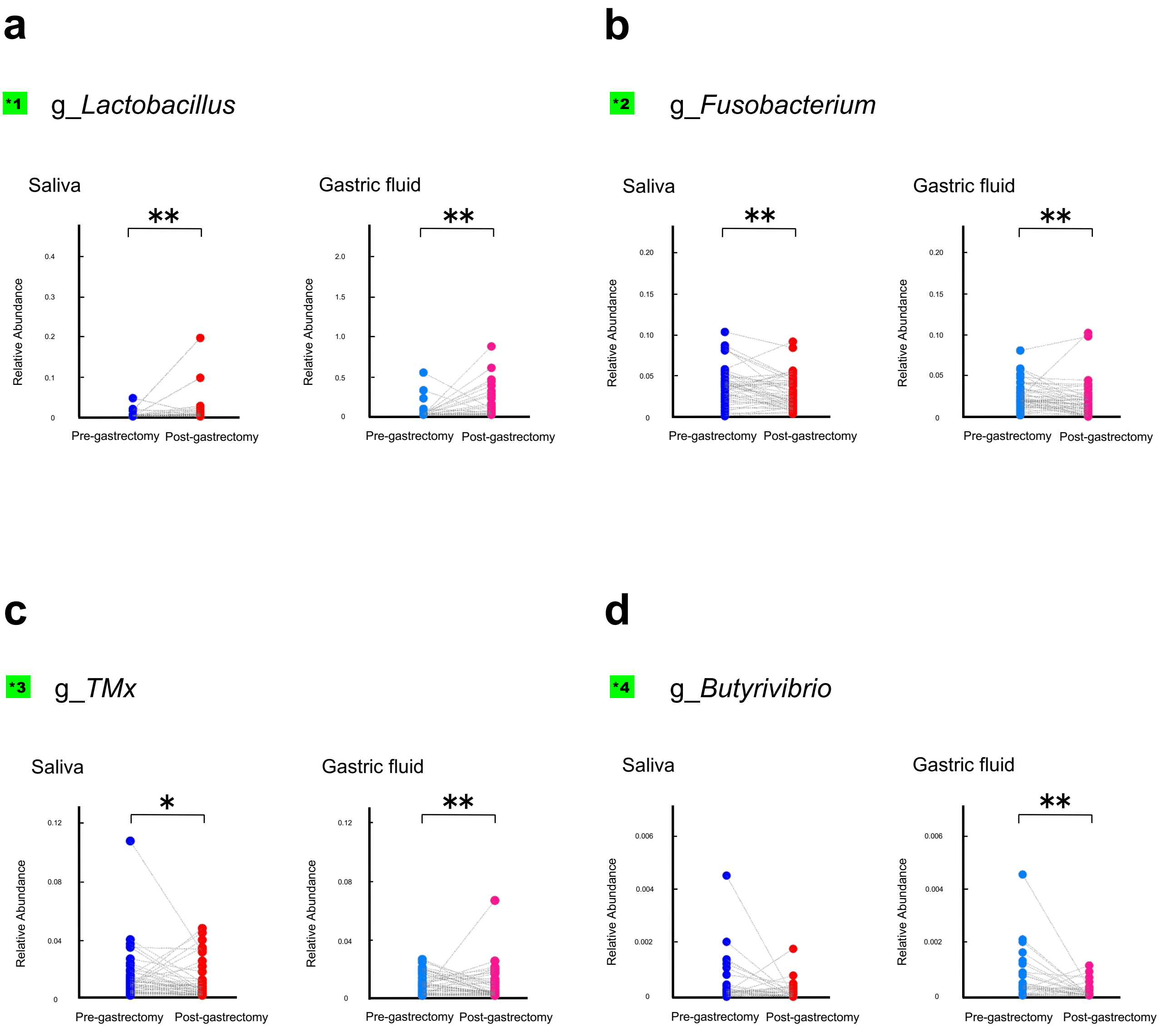

Fig. S7. Changes in the relative abundance of salivary and gastric bacteria with the exclusion of 13 current smokers from the results in Figure 4.

Relative abundance of salivary and gastric bacteria at the time points pre- and post-gastrectomy in pairs of the same individual. (a) Lactobacillus, (b) Fusobacterium, (c) TM7x, and (d) Butyrivibrio.

**, $p < 0.01$; *, $p < 0.05$. Comparisons between the groups were performed using the Wilcoxon signed-rank sum test.
